# Risk of Orthopedic Surgical Site Infections in Patients with Rheumatoid Arthritis Treated with Antitumor Necrosis Factor Alfa Therapy

**DOI:** 10.1155/2012/369565

**Published:** 2012-02-23

**Authors:** Bernardo Matos da Cunha, Licia Maria Henrique da Mota, Leopoldo Luiz dos Santos-Neto

**Affiliations:** ^1^Department of Internal Medicine, Hospital SARAH Brasília, SARAH Network of Rehabilitation Hospitals, SMHS Quadra 301 Bloco A, 70.335-901 Brasília-DF, Brazil; ^2^Department of Rheumatology, University of Brasília, SGAN 605, Av. L2 Norte, 70840-901 Brasília-DF, Brazil; ^3^Department of Internal Medicine, University of Brasília, SGAN 605, Av. L2 Norte, 70840-901 Brasília-DF, Brazil

## Abstract

*Introduction*. International guidelines recommend interruption of anti-TNF medications in the perioperative period, but there are no randomized trials to support such recommendation. *Objectives*. To study literature evidence assessing the risk of surgical site infections in orthopedic surgery patients with RA using anti-TNF drugs, compared to untreated patients or those using conventional DMARD. *Methods*. Systematic review of cohort studies is concerning surgical site infections in orthopedic procedures in patients with RA. *Results*. Three studies were selected. Only one was considered of high-quality, albeit with low statistical power. The review resulted in inconclusive data, since the best quality study showed no significant differences between groups, while others showed increased risk of infections in patients using anti-TNF medications. *Conclusion*. It is unclear whether patients with RA using anti-TNF medications are at increased risk of surgical site infections. Randomized controlled trials or new high quality observational studies are needed to clarify the issue.

## 1. Introduction

Rheumatoid arthritis (RA) affects between 0.2 and 1% of brazilian population [1]. Twenty-five percent of RA patients undergo some surgery for the treatment of orthopedic sequelae after 22 years of followup [2]. Currently, most patients with RA are in use of conventional modifying disease activity drugs (DMARD), and some of them are on antagonists of tumor necrosis factor (anti-TNF) medications [3, 4].

Anti-TNF drugs have been used to treat patients with RA who do not get to remission with one or more conventional DMARD. Systematic reviews have shown no increased risk of bacterial infections after treatment with such drugs [5, 6].

There is no consensus in the literature on the use of immunosuppressive drugs in the perioperative period in orthopedic surgery since there are few studies on the topic. A randomized clinical trial showed no increased perioperative infections in patients on methotrexate [7]. There are no clinical trials assessing treatment with anti-TNF medications in this context so far. The guidelines of the American College of Rheumatology (ACR), British Society of Rheumatology, and Japan College of Rheumatology recommend the suspension of anti-TNF medications in the perioperative period [8–10], but this might lead to worsening of disease activity, which could affect the postoperative rehabilitation.

In observational studies of patients undergoing hip and knee arthroplasties, several independent risk factors for surgical site infection were found, including RA itself, male gender, age greater than or equal to 75 years, secondary osteoarthritis, type of prosthesis, no cement prosthesis, comorbidity index, joint injury by trauma, American Society of Anesthesiology physical status classification greater than or equal to 3, wound hematoma, days of wound drainage, and surgical time, which is the most consistently described risk factor [11–15].

The objective of this study was to perform a systematic review of observational studies on the risk of surgical site infections (SSI) in orthopedic surgery in patients with RA, treated with anti-TNF drugs, compared to untreated patients or those using conventional DMARD.

## 2. Methods

### 2.1. Types of Studies

Prospective or retrospective cohort studies that assessed the risk of SSI in orthopedic surgery in patients with RA, treated with anti-TNF drugs, compared to untreated patients or those using conventional DMARD were eligible. Studies could evaluate patients undergoing any type of orthopedic surgery, including arthroplasty. The minimum followup should be one year, so that all prosthetic infections were accounted [16].

### 2.2. Types of Patients

RA patients are classified according to ACR 1987 criteria [17].

### 2.3. Outcomes

Superficial or deep incisional infections or prosthesis infections, defined by objective criteria.

### 2.4. Search Strategy

We used the following keywords: “anti-tumor necrosis factor,” “DMARD,” “rheumatoid arthritis”, “orthopedic surgery,” and “infection,” all simultaneously and in combinations between themselves. Search was performed in the Cochrane Collaboration, MEDLINE, EMBASE, CINAHL, and LILACS databases. We included only those studies in English, Portuguese, and Spanish.

### 2.5. Data Collection and Analysis of Studies

After the search results, the abstracts were initially assessed. After selecting articles that met inclusion criteria, we performed a general reading of the articles, followed by methodological analysis. Data were collected in a systematic manner on a standardized form. Initially, it was noted whether the study was prospective or retrospective and whether there was sample size calculation. Then, we applied Newcastle-Ottawa Cohort Quality Assessment Scale [18], which has good applicability for the purposes of this review. These criteria split the analysis into three areas: selection, comparability, and outcome. In each of these areas, we applied a number of questions and, according to the answer, a “asterisk” is attributed. In the fields “selection” and “outcome,” it is possible to assign one “asterisk” for each question, while it is possible to assign two “asterisks” to the question “comparability.” We performed an adaptation to questions so that they could apply to the scenario of this review. The assessment details are shown in Table 1. Studies were considered of high quality if they had at least one “asterisk” in each area, and the sum of the “asterisks” were equal to or greater than five.Due to heterogeneity of the studies, no qualitative data analysis was performed.

## 3. Results

### 3.1. Search

**Figure 1:**
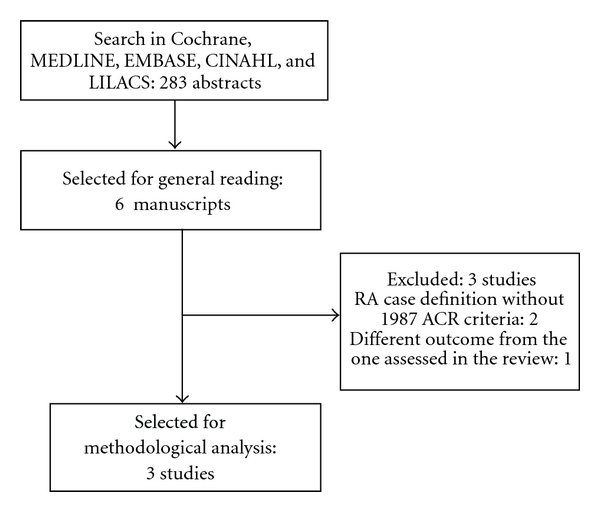
Fluxogram of studies selection. Acronyms: RA: rheumatoid arthritis; ACR: American College of Rheumatology.

Initially 283 abstracts were found in MEDLINE. Search in other databases did not add additional abstracts. Six abstracts were selected according to inclusion criteria and, after general manuscript reading, three studies [19–21] were included in the review. Details of articles selection are shown in Figure 1. Since we selected few articles, it was possible to describe each one separately.

### 3.2. Description of Studies

Studies included 1767 procedures. The study by Momohara et al. was not clear about the number of of patients, so was not possible to express their exact number.

The study by den Broeder et al. [19] is a retrospective cohort that included 1219 patients in 768 procedures from two centers in the Netherlands. Its population consisted of patients who underwent various types of orthopedic surgery between 2001 and 2004. Procedures were excluded if the time from the last procedure was less than three months. Patients were divided into two groups: those who were on anti-TNF therapy (cohort 2) versus those who had never used these medications (cohort 1), but it was not clear which DMARD they were in use. The first group was then divided into two groups: patients who were in use of anti-TNF therapy in the perioperative period (cohort 2b) versus those who had discontinued the drug at least four half-lives before the surgery (cohort 2a). In cohort 2, patients were being treated with infliximab in 80 procedures, etanercept in 79, and adalimumab in 37 procedures. In addition to the primary outcome, this study also evaluated the incidence of wound dehiscence, bleeding or hematoma, subluxation, reoperation, and death. The rate of SSI in cohorts 1, 2*ª*, and 2b was 4.0%, 5.8% and 8.7%, respectively. No increased risk of SSI was found in cohort 2, compared to cohort 1. In this comparison, odds ratio (OR) values and confidence intervals (CI) were not provided and were calculated by the main author of this review (OR 1.84, 95% CI 0.98 to 3.44). The comparison between the cohorts 2a and 2b showed similar numbers of SSI between groups (OR 1.56, 95% CI 0.52 to 4.66). Regarding the “wound dehiscence” outcome, there was an increased incidence in patients who continued using the anti-TNF compared with those who had stopped (OR 11.2, 95% CI 1.4 to 90). Comparsion between patients on anti-TNF therapy who discontinued the drug and patients who were anti-TNF naive showed a reduced incidence in the former group (OR 2.4, 95% CI 1.1 to 5.0). There were no data on disease activity.

The study by Kawakami et al. [20] is a retrospective cohort that included 128 procedures in 112 patients from a single center in Japan. The population consisted of patients undergoing joint surgery between 2004 and 2009, in which most of them were arthroplasties. A 1 : 1 matching was performed among patients receiving anti-TNF versus conventional DMARD. In the group on anti-TNF therapy, patients were taking infliximab in 35 surgeries and etanercept in 29 surgeries. Patients on conventional DMARD were using methotrexate in 48 cases, sulfasalazine in 18 cases, bucillamine (an immunomodulator drug developed in Japan, similar to D-penicillamine) in 6 cases, and D-penicillamine in 4 cases, either alone or in combination. In addition to the primary outcome, the presence of arthralgia and deep vein thrombosis (DVT) diagnosed with ultrasonography was assessed, but without specifying whether tests were performed in all patients. Anti-TNF medications were discontinued 2–4 weeks before surgery and it is unclear if conventional DMARD were also discontinued. OR and CI were not provided and were calculated by the main author based on information collected in the study. The SSI rate was higher among patients on anti-TNF therapy than in patients receiving conventional DMARD (12.5%  and 1.6% resp.) (OR 9.0, 95% CI 1.1–74.22). The incidence of DVT was higher among patients on anti-TNF therapy than in patients receiving conventional DMARD (OR 2.9, 95% CI 1.2 to 6.9). Regarding the outcome “arthralgia,” comparisons were made only within the group of patients treated with anti-TNF medications.The study of Momohara et al. [21] is a retrospective analysis of a prospective cohort that included 420 procedures performed by the same group of Kawakami et al. The population consisted of patients undergoing hip (81 cases) and knee (339 cases) arthroplasties between 2005 and 2009. Initially, the authors divided patients into two groups: individuals on anti-TNF therapy versus those using conventional DMARD. However, when reporting the results, the authors have chosen to make comparisons according to the outcome, setting a nested case-control design. As the study provided the data for each group, it was possible to calculate OR and CI for the comparison according to the risk factor. In the group of patients using anti-TNF medications, 19 patients were treated with infliximab, 23 with etanercept, and 2 with adalimumab. In the group of patients using conventional DMARDs, 279 patients were treated with methotrexate, 93 with sulfasalazine, 52 with bucillamine, 7 with minocycline, 4 with leflunomide, 31 with tacrolimus, 15 with mizoribine (a drug with immunomodulatory mechanism of action similar to mycophenolate mofetil), 3 with cyclophosphamide, 9 with actarit (an immunomodulator drug developed in Japan, a nitric oxide inhibitor), 4 with auranofin, 1 patient with aurothiomalate, and 16 patients with D-penicillamine, alone or in combination. Conventional DMARD were kept during the perioperative period, and anti-TNF medications were discontinued 2–4 weeks before surgery. The SSI rate was higher among patients using anti-TNF than in patients receiving conventional DMARD (20.8% and 4.0% resp.) (OR 6.3, 95% CI 2.6 to 14 9). Most infections were superficial, and there was no data on disease activity.

### 3.3. Quality Rating

The only study considered of high quality by our assessment was AA den Broeder et al. (Table 2). It was also the only study that included patients receiving adalimumab and assessed allocation bias. However, the study did not achieve sufficient statistical power to detect small differences. A *post hoc* calculation of statistical power of this study was 49.4%. The other two studies showed several methodological flaws and heterogeneous methodologies, which hampered the statistical analysis between groups with and without the use of anti-TNF medications, including logistic regression, so it is likely to have occurred association bias. On the other hand, in these studies, anti-TNF medications were discontinued 2–4 weeks before surgery, which may have diminished the risk of infections with such drugs. As followup was not informed, it is not clear whether all infections were recorded. There was no information about the frequency of progression to deep infections.

## 4. Discussion

Currently, there are no randomized trials that have assessed safety of anti-TNF medications in the orthopedic surgery perioperative period. The available body of evidence is based on observational studies and expert opinion. Although some international guidelines recommend discontinuation of medications before surgery, according to drug half-life [6–8], the results of this review indicate that there is insufficient evidence to support these recommendations. Two Japanese studies, performed by the same group, have shown significantly increased risk of surgical site infections in patients on anti-TNF therapy when compared to patients using conventional DMARD, but it is not clear if both studies included some patients in common. In contrast, the single high quality study (den Broeder AA et al.), performed in another ethnic group, showed no increased risk of infections. Moreover, it was the only study that compared anti-TNF naïve patients to the ones in current treatment with anti-TNF medications and to others who discontinued the anti-TNF drugs before surgery, including patients receiving adalimumab. It was also the only study to describe follow-up time. Unfortunately, this study had a low statistical power, probably because the estimated number of infections was less than expected. In the Japanese studies, anti-TNF drugs were discontinued in the perioperative period, not allowing the observation of outcomes in the presence of full serum levels of the analyzed drugs.

On the other hand, none of the studies properly assessed disease activity. Discontinuation of immunomodulatory treatment may allow the reactivation of joint inflammatory activity in the perioperative period, what can lead to difficulties in the rehabilitation process.

In conclusion, it is not clear, according to the body of evidence currently available, whether patients with RA using anti-TNF medications are at increased risk of surgical site infections, compared to patients receiving conventional DMARD. Multicenter randomized controlled trials or new prospective high quality observational studies are needed to make it possible to reach a firm conclusion, including patients from distinct ethnicities, with sufficiently numerous population and assessing adalimumab use. For now, we recommend that discontinuation of anti-TNF drugs should occur after a case-based discussion between clinicians and surgeons, considering the risks and benefits, taking into account patient characteristics, the intended procedure and the institution where the surgery will be performed.

## Figures and Tables

**Table 1:** Newcastle-Ottawa Cohort Quality Assessment Scale, adapted for review purposes, as description from authors. Acronym: RA: rheumatoid arthritis.

Selection
(1) Representativeness of the exposed cohort
(a) truly representative of the average RA patient in the
community*
(b) somewhat representative of the average RA patient in the
community*
(c) selected group of users e.g., nurses, volunteers
(d) no description of the derivation of the cohort
(2) Selection of the nonexposed cohort
(a) drawn from the same community as the exposed cohort*
(b) drawn from a different source
(c) no description of the derivation of the nonexposed cohort
(3) Ascertainment of exposure
(a) secure record (e.g., surgical records)*
(b) structured interview *
(c) written self-report
(d) no description
(4) Demonstration that outcome of interest was not present at start
of study
(a) yes*
(b) no

Comparability
(1) Comparability of cohorts on the basis of the design or analysis
(a) study controls for surgical time*
(b) study controls for any additional factor *

Outcome
(1) Assessment of outcome
(a) independent blind assessment*
(b) record linkage*
(c) self-report
(d) no description
(2) Was followup long enough for outcomes to occur?
(a) yes (1 year)
(b) no
(3) Adequacy of followup of cohorts
(a) complete follow up—all subjects accounted for*
(b) subjects lost to followup unlikely to introduce bias—small
number lost—>80% follow up, or description provided of those
lost*
(c) followup rate < 80% and no description of those lost
(d) no statement

*Studies were considered of high quality if they had at least one asterisk in each area, and the sum of the asterisks were equal to or greater than five.

**Table 2:** Quality of studies according to Newcastle-Ottawa Cohort Quality Assessment Scale.

Studies	Selection	Comparability	Outcome	Total	Quality
den Broeder AA e cols.	****	**	***	9	high
Kawakami K e cols.	****			4	low
Momohara S e cols.	****			4	low

*Studies were considered of high quality if they had at least one asterisk in each area, and the sum of the asterisks were equal to or greater than five.
